# Patient Satisfaction and Cosmetic Outcome in a Randomized, Prospective Study of Total Knee Arthroplasty Skin Closure Comparing Zip Surgical Skin Closure with Staples

**DOI:** 10.7759/cureus.6705

**Published:** 2020-01-19

**Authors:** Bruce Menkowitz, Gerilyn Olivieri, Ori Belson

**Affiliations:** 1 Orthopedic Surgery, Einstein Medical Center Montgomery, East Norriton, USA; 2 Mechanical Engineering - Medical Studies, Cornell University, Ithaca, USA

**Keywords:** arthroplasty, patient satisfaction, pain, staple, scar, zip, zipline

## Abstract

Background

Evaluation of the Zip surgical skin closure device (Zip) versus metal staples regarding patient satisfaction and cosmesis after total knee arthroplasty (TKA).

Methods

Patients undergoing TKA at Einstein Medical Center Montgomery were randomized to receive skin closure using Zip or staples. Patient satisfaction was assessed by surveys at discharge, three weeks and six weeks post-operatively. Cosmesis according to patients and observers was assessed using the Patient and Observer Scar Assessment Scale (POSAS).

Results

Randomization resulted in 21 Zip and 20 staple patients. Two subjects were lost to follow-up and one patient suffered cardiac arrest. Surgeon satisfaction favored Zip over staples on day of surgery and patient discharge. At three weeks, Zip was favored over staples for patient-reported movement and device removal pain. Patient satisfaction results favored Zip for comfort, ease of wound care, and hospital selection based on wound closure. POSAS favored Zip for appearance, pain, itching, color, stiffness, thickness, irregularity, vascularity, pigmentation, relief, pliability, surface area, and observer opinion. Subject opinion resulted in no difference between groups. At six weeks, no differences were found for patient-rated movement pain or ease of wound care. POSAS favored Zip for color, stiffness, thickness, vascularity, pigmentation, thickness, relief, pliability, surface area and observer opinion.

Conclusion

Satisfaction with the closure method and patient and physician assessments of cosmesis were superior with Zip. Orthopedic surgeons strive to optimize TKA patient satisfaction. Skin closure can influence patient satisfaction as the memory of their recovery fades and the scar remains the most visible reminder of their experience.

## Introduction

Total knee arthroplasty (TKA) is well established as an effective treatment to reduce pain and restore function for patients with severe osteoarthritis. Advances in prosthetic technology and surgical technique have contributed to improved functional outcomes and lower post-surgical pain as well as a reduction in surgery-related complications [1-5]. The selection of skin closure for TKA can be a significant contributor to maximizing patient satisfaction, in the first few months after surgery as well as beyond, as the patient's memory of the surgical recovery fades and the scar remains the most visible reminder of their experience. Studies have compared traditional methods such as staples and sutures with respect to pain, wound care and scar quality. However, patient satisfaction regarding suture or staple closure is currently not supported by any clear evidence [6,7]. This study sets out to evaluate the effect, if any, on patient satisfaction of a new, non-invasive method of skin closure in comparison to traditional metal staples.

## Materials and methods

This prospective, single-center, randomized clinical trial was conducted at Einstein Medical Center Montgomery in East Norriton, PA, USA. The Institutional Review Board at Einstein Healthcare Network approved the study and it was then registered on ClinicalTrials.gov with an identifier code of NCT03178266. The methods were not altered after the trial began.

Patients aged 18-80 years undergoing primary elective total knee arthroplasty via midline knee skin incision were included. During their presurgical visits, all included patients provided informed consent to physicians. We excluded patients that do not meet the conditions listed in the wound closure device warnings, precautions, and contraindications, patients with comorbidities or conditions that the investigator deems to be ineligible for the study or patients without the capacity to give informed consent (e.g., dementia).

All surgeries were conducted by the same surgical team. Before the surgery, each patient received skin preparation with chlorhexidine solution. Subcutaneous tissue was closed with absorbable interrupted suture. Patients were randomly assigned to one of two conditions. In one, the patients’ skin incisions were closed with the Zip® Surgical Skin Closure device (ZipLine Medical, Inc., Campbell, CA). In the other, the patients’ skin incisions were closed with nonabsorbable metal staples. The staples were then removed between 12 and 15 days after the surgery. When the staples were removed from the group who received staple closure, Steri-Strips (3M, St. Paul, MN) were placed on the patients’ incision. These patients were told to remove the Steri-Strips after seven days. No Steri-Strips were placed on the patients who received the Zip device.

**Figure 1:**
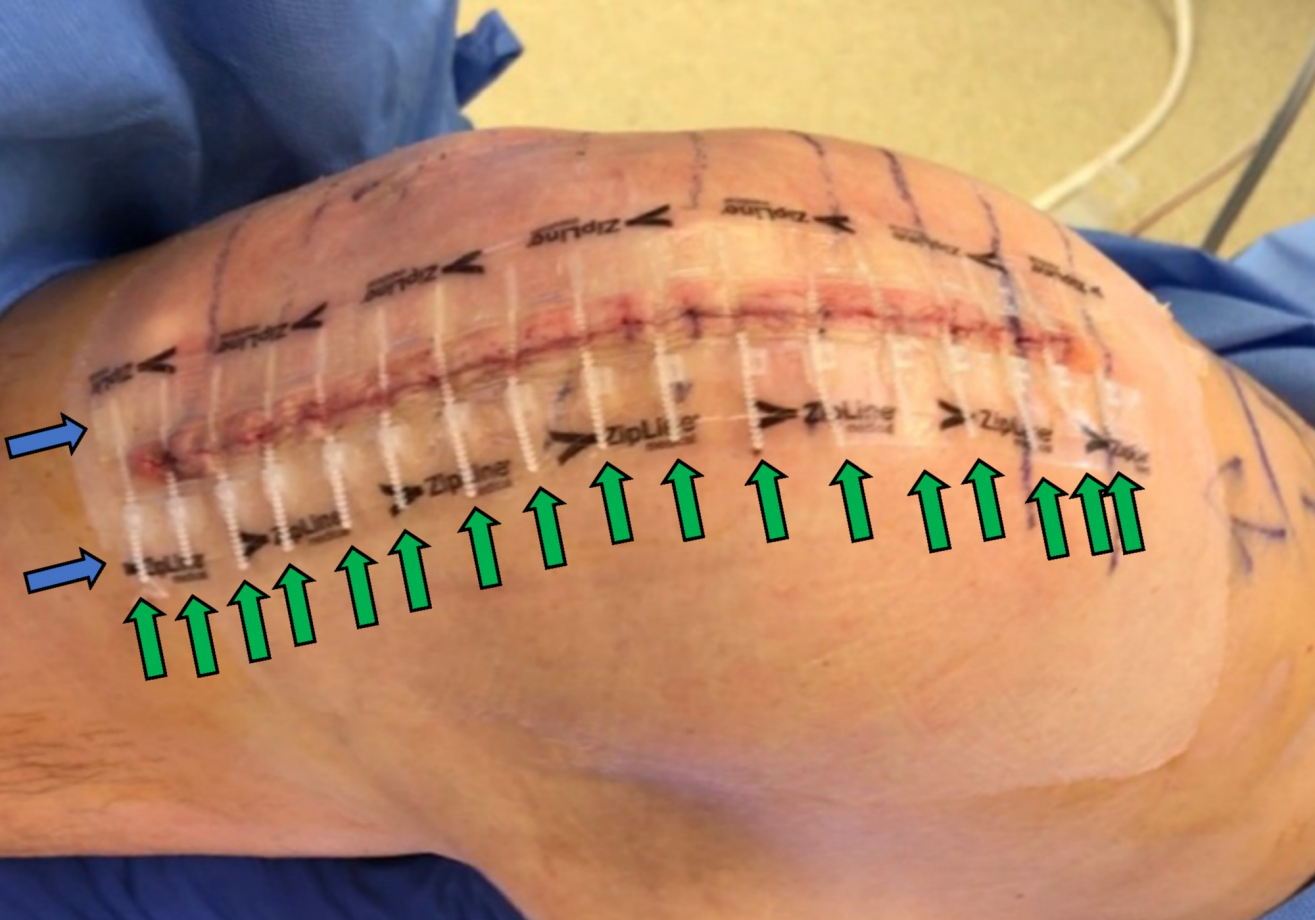
Zip surgical skin closure device on total knee arthroplasty, ZipLine Medical, Inc., Campbell, CA, USA. Blue arrows indicate polyurethane strips and green arrows indicate nylon zip-tie-type ratcheting straps.

The Zip device shown in Figure 1 is a non-invasive wound closure device consisting of two polyurethane strips attached to healthy, intact skin on either side of an incision with a hydrocolloid pressure-sensitive skin adhesive. Closure is achieved by means of a series of interconnected nylon zip-tie-type ratcheting straps, with the terminus of each pair of locks and straps interconnected along each polyurethane strip with a force-distributing longitudinal nylon strut. The device enables adjustable closure at different force levels along the wound, and the longitudinal nylon struts distribute closing force from the straps to the adhesive strips between each strap. A novel feature of the adjustment mechanism allows loosening the strap if too much tension is applied during closure. Levi et al. demonstrated that this apparatus provides greater shielding of the wound from perturbation caused by distraction forces than intradermal sutures [8]. Carli et al. demonstrated a reduction in post-discharge wound care with the Zip device [9]. Safa et al. demonstrated how the Zip device may reduce bacterial penetration in a wound compared to sutures [10]. Koerber et al. demonstrated significant reduction in procedure time with the Zip device [11]. Our aim was to determine what, if any, impact this device may have on patient satisfaction.

Randomization was achieved in a computer-generated 1:1 ratio. An opaque envelope, which was numbered sequentially, was opened at the time of surgery for each enrolled subject. Patient satisfaction regarding the method of incision closure was the primary endpoint. At hospital discharge patients were asked to score their satisfaction with the wound closure using a five-point Likert scale. Patients returned to clinic for wound closure device removal at three weeks postoperatively and were asked a more extensive set of incision-related questions using the Likert scale regarding pain, comfort, wound care, anxiety towards device removal and overall satisfaction with the wound closure method used. In addition, during the three-week visit both patient and provider completed the respective portions of the Patient and Observer Scar Assessment Scale (POSAS). The POSAS consists of two components. One of these is a Patient Scar Assessment Scale (PSAS), completed by the patient. The other is a component called the Observer Scar Assessment Scale (OSAS), completed by the observer. The following six items are evaluated by the PSAS: color, pain, stiffness, itchiness, irregularity and thickness. Five items are evaluated by the OSAS: thickness, pigmentation, vascularity, pliability and relief. A ten-point scale is utilized to score each item. Specifically, this scale compares the patients’ skin to normal skin (normal skin = 1). Then, the score is summed separately for OSAS (range 550) and PSAS (range 660). This value is sometimes totaled to produce what is called the POSAS score (range 11,110). The lower the score, the closer the patient’s skin resembles normal skin. At six weeks postoperatively, patients returned again to clinic and completed a similar Likert scale questionnaire (omitting questions regarding device removal), and both subjects and provider completed the respective portions of the POSAS questionnaire.

Based on previous research, a statistical power analysis was performed for sample size estimation (Poster: Benner RW, Behrens JP. A Novel Skin Closure Device for Total Knee Arthroplasty: Randomized Controlled Trial versus Staples. American Association of Hip and Knee Surgeons; Nov 3, 2017). To detect a 20% difference in patient satisfaction with a significance level set to 0.05 and a power set to 0.9, 20 samples per group were required.

The primary purpose of the study per the Clinical Investigational Plan (CIP) protocol was to evaluate patient satisfaction of closure methods for comparisons between staple and Zip device closure methods after joint knee arthroplasty. The satisfaction data was gathered by both POSAS Scale (Patient and Observer) data, and overall Patient Satisfaction data. The primary and secondary endpoints were to assess the POSAS data at six weeks post-operative procedure, and assessment of subject experience and satisfaction results, respectively. The final sample (n) sizes at the three-week (mid-interval) were 18 patients for staples, and 21 patients for Zip device closure methods. At the six-week data collection endpoint, satisfaction and POSAS data was collected from a final sample size of 17 patients with staples and 20 patients with the Zip closure device.

## Results

Demographics

Enrollment occurred from December 2017 to March 2018. Of the 41 subjects enrolled, randomization resulted in 21 subjects receiving the Zip device and 20 subjects receiving staples for skin closure. Early termination occurred with three subjects: two subjects were lost to follow-up and one subject withdrew due to an adverse event not related to the study.

Subject demographic data of both groups are shown in Table 1. There were no significant differences in terms of age, gender, BMI, or skin type classification.

**Table 1:** Subject demographic data. BMI: Body Mass Index; *Skin type was measured using the Fitzpatrick scale. The Fitzpatrick scale measures skin as type 1 when the skin always burns, type 2 when the skin usually burns, type 3 when there is sometimes a mild burn, type 4 when the skin rarely burns, type 5 when the skin very rarely burns and type 6 when the skin never burns.

	Zip mean (standard deviation)	Staple mean (standard deviation)
Age	66.3 (7.41)	64.7 (6.78)
Gender	70% female	52% female
BMI	32.1 (5.46)	35.8 (9.36)
Skin Type*	2.90 (1.25)	3.24 (1.37)

Day of surgery results

After the procedure, surgeons were asked to rate their satisfaction with the wound closure method using a 5-point Likert scale (0 = Very Satisfied, 5 = Very Dissatisfied). A hypothesized t-test of difference = 0 (vs ≠ 0) indicated that use of the Zip device was preferred over staples (p = 0.045).

Hospital discharge results

At hospital discharge, surgeons were again asked to rate their satisfaction with the wound closure method using the Likert scale. Results indicated a preference for the Zip device over staples (p = 0.045).

Three-week follow-up results

**Figure 2:**
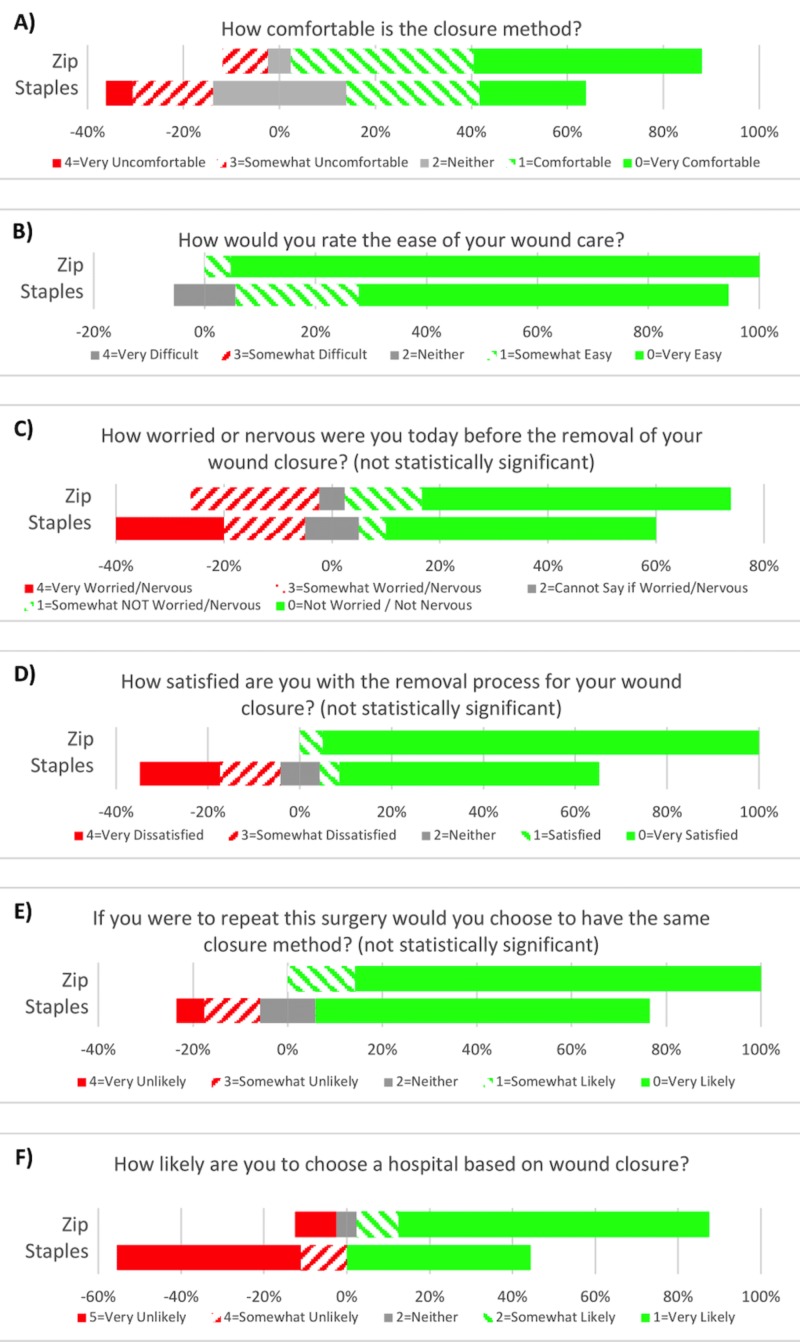
Three-week patient satisfaction results from Likert scale questions.

During the three-week follow-up visit, patients were asked six questions relating to patient satisfaction using a Likert scale. All results favored the Zip device over staples, with statistical significance reached for comfort (p = 0.03), ease of wound care (p = 0.03), and likelihood to select a hospital based on their wound closure type used (p = 0.01). This is illustrated in Figure 2.

**Figure 3:**
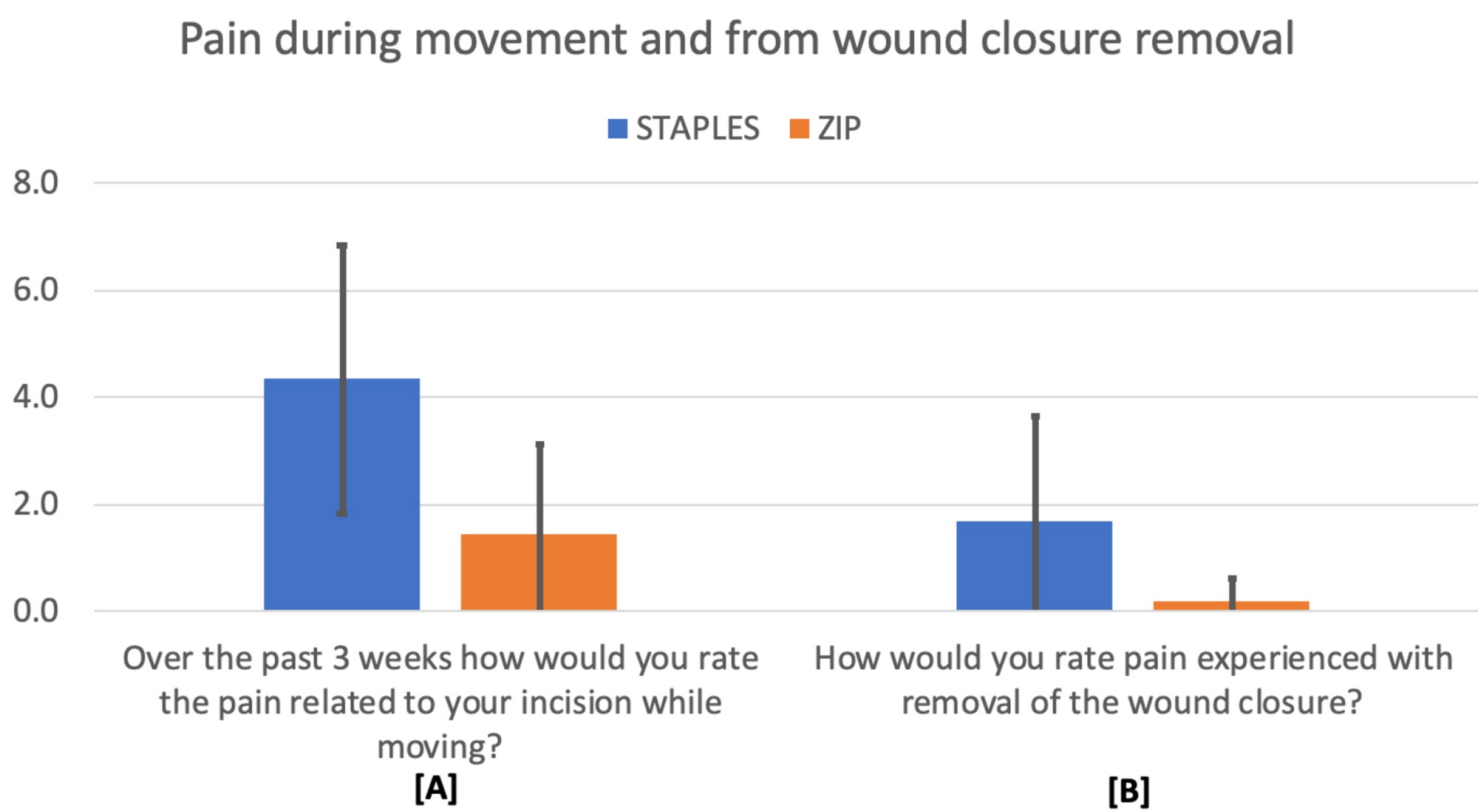
Three-week pain (average and standard deviation) from movement and wound closure removal. Statistically significant results. 0 = no pain, 10 = worst pain.

**Figure 4:**
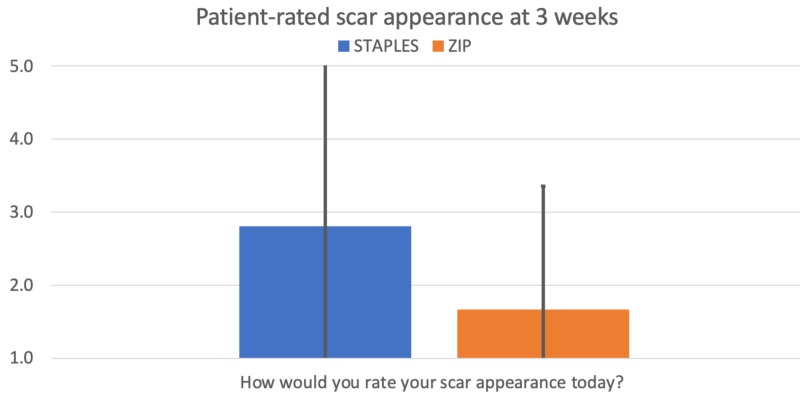
Patient-reported scar appearance at three weeks. 1 = minimal scar, 5 = significant scar.

Pain during movement (p = 0.0003) and removal (p = 0.006) was assessed using a 10-point scale, with both results achieving statistical significance favoring the Zip device. This is illustrated in Figure 3. Patients also rated the appearance of their scar at three weeks using a 5-point scale, with the Zip scar favored over staples with statistical significance (p = 0.010). This is illustrated in Figure 4.

**Figure 5:**
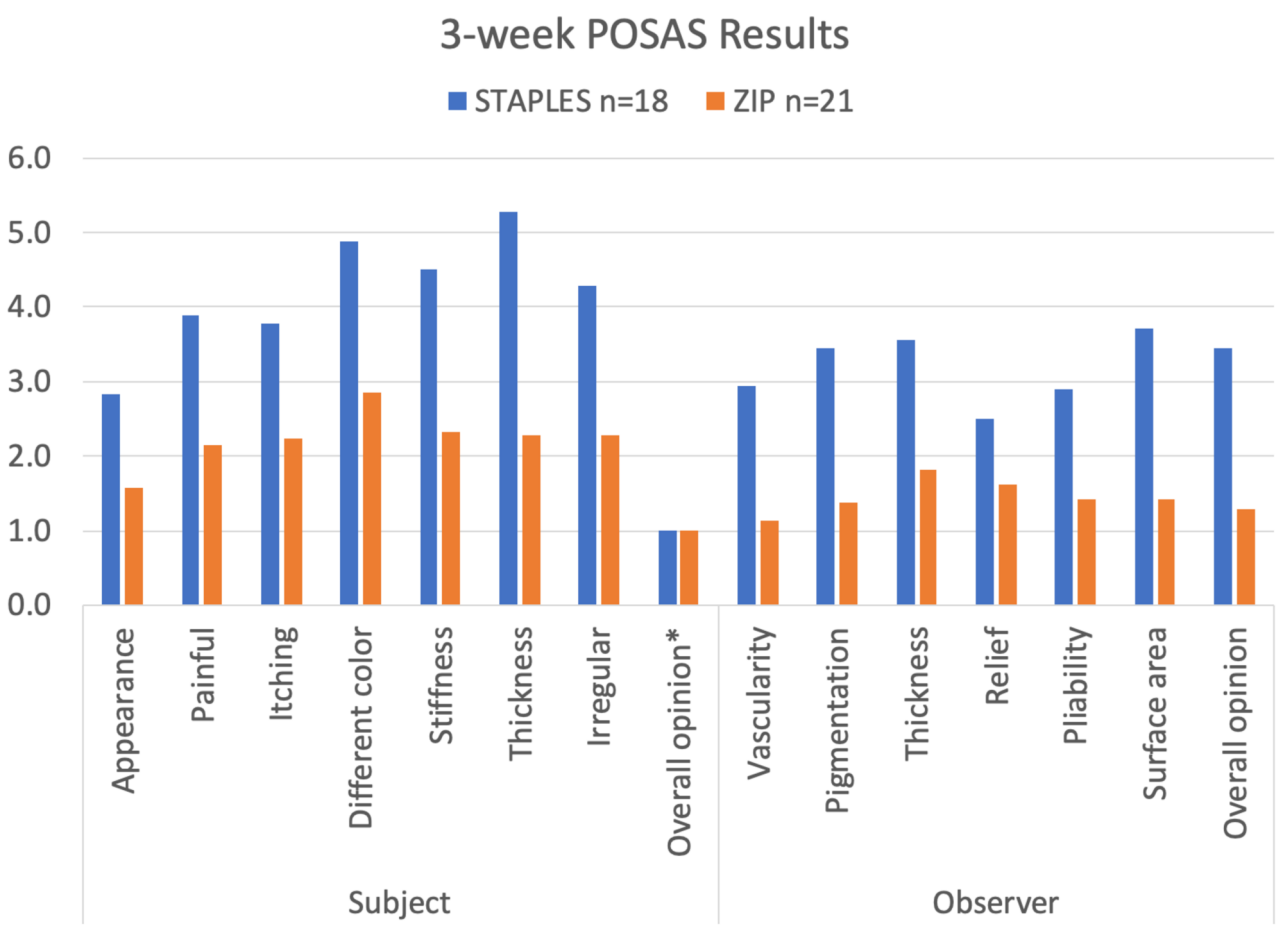
Three-week POSAS results. Lower scores represent results that are closest to normal skin. *Denotes a non-statistically significant result; POSAS: Patient and Observer Scar Assessment Scale.

With the exception of subject overall opinion, which resulted in equally positive responses for both arms, POSAS Scale (Observer and Patient) attributes achieved statistical significance in favor of the Zip device for appearance (p = 0.046), pain (p = 0.050), itching (p = 0.032), color (p = 0.022), stiffness (p = 0.002), thickness (p = 0.001), irregularity (p = 0.006), vascularity (p = 0.0003), pigmentation (p = 0.001), relief (p = 0.015), pliability (p = 0.003), surface area (p = 0.0001), and observer overall opinion (p = 0.0001). This is illustrated in Figure 5.

Six-week follow-up results

At six weeks, no significant differences between the two groups were found for patient-rated pain during movement or ease of wound care.

**Figure 6:**
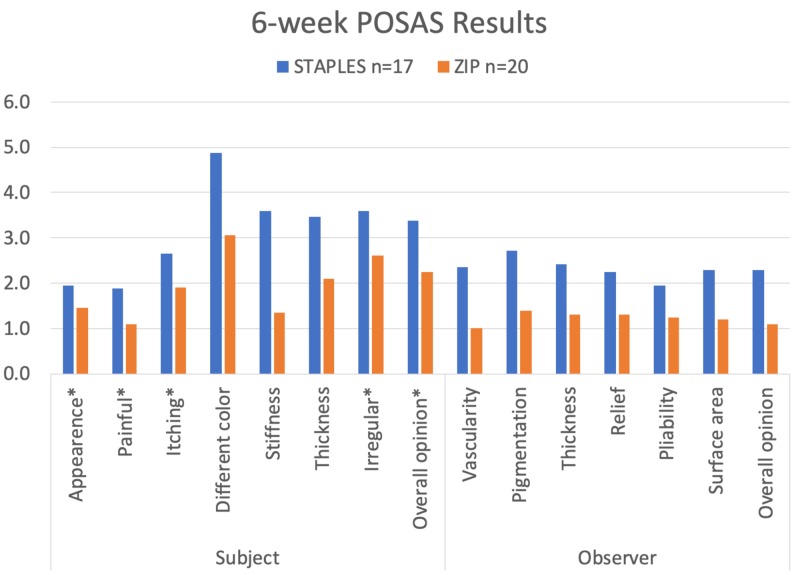
Six-week POSAS results. *Denotes a non-statistically significant result; POSAS: Patient and Observer Scar Assessment Scale.

POSAS Scale results favored the Zip device over staples, achieving statistical significance for color (p = 0.021), stiffness (p = 0.002), thickness (p = 0.048), vascularity (p = 0.002), pigmentation (p = 0.003), thickness (p = 0.003), relief (p = 0.001), pliability (p = 0.005), surface area (p = 0.002) and observer overall opinion (p = 0.0004). This is illustrated in Figure 6.

## Discussion

Staples are typically selected to close surgical incisions due to their fast application and reliability. However, staples remain an imperfect solution due to their invasiveness, increased rate of wound complications, limitation on knee range of motion and greater patient pain (Poster: Benner RW, Nov 3, 2017) [9]. In this study, the Zip device has shown to be a superior replacement for staples, providing rapid application, secure, adjustable closure, greater patient comfort and range of motion. In addition, the non-invasive Zip device offers advantages that include no need for staple or suture removal, no risk of needlestick injury, absence of abscesses that can occur from absorbable suture material and elimination of tissue punctures that can be pathways for bacteria [9,10,12].

The ziptie-like straps on the Zip device enabled very precise tensioning of the incision, which may have contributed to the favorable results shown here. While staples tended to compress the incision and blanch the skin between the staple puncture points due to the non-adjustable compression when the staple gun is squeezed, we found that with the Zip device it was very easy to achieve approximation without resulting in an ischemic condition that could impact healing and scarring. Although closure time was not measured in the study, we found that application of the Zip device took about the same amount of time as applying metal staples to the incision. There were no observed skin reactions to the adhesive used on the Zip device.

## Conclusions

Compared to those whose incisions were closed with staples, the patients whose incisions were closed with Zip surgical skin closure had superior rankings regarding the following measures: satisfaction with the scar’s appearance, satisfaction with the method of closure, and patient and physician satisfaction with scar cosmesis. While significant gains have been achieved in improving functional outcomes for TKA, surgeons continue to pursue ways to optimize patient satisfaction. The selection of the skin closure is a significant contributor to maximizing patient satisfaction, in the first few months after surgery as well as beyond. While the memory of the surgical recovery fades, the scar is a visible reminder of their experience. Providing patients and surgeons with options ensures that each patient receives the best possible surgical and cosmetic outcome.
